# Quality of life in Norwegian pregnant women and men with pregnant partners, and association with perception of sleep and depressive symptoms: a cross-sectional study

**DOI:** 10.1186/s12884-023-05379-x

**Published:** 2023-01-18

**Authors:** Malene Brekke, Amin Amro, Milada Cvancarova Småstuen, Kari Glavin, Beate Solberg, Anne-Martha Utne Øygarden, Kristin Marie Sæther, Trude Haugland

**Affiliations:** 1 VID Specialized University, Oslo, Norway; 2 Oslo Metropolitan University, Oslo, Norway

**Keywords:** Quality of life, Pregnancy, Women, Men, Depressive symptoms, Sleep

## Abstract

**Background:**

Pregnant women and men with pregnant partners experience variations in quality of life (QoL) during pregnancy, a period characterized by physical, psychological, and social changes. Pregnancy is associated with reduced QoL, depressive symptoms, and sleep problems. This study aimed to: (1) determine whether Norwegian pregnant women and men with pregnant partners differed in QoL levels in the third trimester of pregnancy; (2) determine whether the relationship between perception of sleep and QoL is moderated by depressive symptoms, when analyzed separately in pregnant women and men with pregnant partners; and (3) determine whether selected possible predictive factors were associated with QoL when stratified by level of depressive symptoms, in pregnant women and men with pregnant partners separately.

**Methods:**

A cross-sectional study conducted between October 2018 and January 2020 included 228 pregnant women and 197 men with pregnant partners in the third trimester of pregnancy. The age range was 22–50 years. QoL was assessed using the World Health Organization Quality of Life Questionnaire brief version, depressive symptoms using the Edinburgh Postnatal Depression Scale, and perception of sleep by a single item. Data were analyzed in SPSS version 28 using descriptive statistics, the PROCESS macro for moderation analyses, and multivariate linear regression. The level of statistical significance was *p* < 0.05.

**Results:**

Pregnant women reported significantly lower QoL scores on the physical health and psychological domains than the men with pregnant partners. Our data did not reveal any moderating effect of depressive symptoms on the relationship between the perception of sleep and QoL. Depressive symptoms in the pregnant women were found to be a significant predictor of lower QoL in all domains. In the men with pregnant partners, getting enough sleep was a significant predictor of higher QoL in all domains. In the pregnant women without depressive symptoms, higher QoL in the physical health domain was significantly associated with the perception of getting enough sleep.

**Conclusion:**

Women in the final trimester of pregnancy experience poor QoL compared to the men with pregnant partners. Pregnant women with depressive symptoms have lower QoL compared to those without depressive symptoms. The perception of getting enough sleep was associated with better QoL.

## Background

Pregnant women and men with pregnant partners experience physical, psychological, and social changes during pregnancy and the transition to parenthood [1, 2]. Measures of Quality of Life (QoL) are often used to explore patient outcomes along physical, psychological, and social dimensions [3, 4]. The World Health Organization (WHO) defines QoL as: “… individuals’ perceptions of their position in life in the context of the culture and value systems in which they live and in relation to their goals, expectations, standards, and concerns” [5]. This broad definition acknowledges QoL as a subjective perception and multidimensional concept [5]. Individuals have different expectations of health and life, and individuals in similar situations may appraise their QoL differently [6]. Health is defined by WHO as “a state of complete physical, mental and social well-being and not merely the absence of disease or infirmity” [7]. Hence, pregnant women and men with pregnant partners may experience physical, psychological and social changes as health-related challenges, such as sleep problems and depressive symptoms, affecting QoL [8, 9].

The QoL of pregnant women is identified as lower than in the general population [10] and decreasing throughout pregnancy trimesters [8, 9]. A decrease in QoL is linked mainly to the physical domain [8, 9]. The QoL of men during their partner’s pregnancy is also found to decrease [11, 12], but male partners’ QoL is higher than in pregnant women, especially in the physical domain [12, 13].

Women experience challenges related to sleep throughout pregnancy [14, 15], challenges that are associated with lower QoL in women during pregnancy [10, 16, 17]. Richter et al. [18] found no decrease in sleep satisfaction for men across the three trimesters of their partner’s pregnancy. Depressive symptoms are prevalent in pregnant women and in men with pregnant partners during pregnancy [19, 20]. In pregnant women, depressive symptoms are associated with lower QoL [10, 16, 21, 22] and problems with sleep [23, 24]. Men with a pregnant partner and with poor sleep quality report significantly higher scores of depressive symptoms [25].

We found no studies exploring the interaction between sleep and depressive symptoms and its effect on QoL. The QoL of Norwegian pregnant women and men with pregnant partners, and its associated factors, have been sparsely explored. Measuring the outcomes of the physical, psychological and social changes in pregnant women and men with pregnant partners during pregnancy can provide insights into their perspectives of this period [26]. Based on the current state of the science, we hypothesize that depressive symptoms affect the strength of the relationship between QoL and the perception of sleep, and that the level of depressive symptoms moderates the effect of the perception of sleep on QoL.

Hence, the aims of this study were: 1) to determine whether Norwegian pregnant women and men with pregnant partners differed in QoL levels in the third trimester of pregnancy; 2) to determine whether the relationship between perception of sleep and QoL is moderated by depressive symptoms, when analyzed separately in pregnant women and men with pregnant partners; and 3) to determine whether selected possible predictive factors were associated with QoL when stratified by level of depressive symptoms, in pregnant women and men with pregnant partners separately.

## Methods

### Study design

This is a cross-sectional study and sub-study of the New Families (NF) research project, a prospective non-randomized controlled study. The NF research project is registered at clinicaltrial.gov (identifier: *NCT04162626*) and approved by the Regional Committees for medical and health research ethics in Norway (reference no: 2018/1378) and the Norwegian Centre for Research Data (project no: 735207).

The Strengthening the Reporting of Observational Studies in Epidemiology (STROBE) checklist for cross-sectional studies [27] was followed for the reporting of this study.

### Setting and participants

The setting of this study was clinics in the Norwegian Child Health Services, including the prenatal care provided to pregnant women by midwives. Pregnant women were screened for eligibility and invited to participate in the study by the midwife or the clinic secretary, when attending pregnancy check-ups in five districts in the city of Oslo, from October 2018 to December 2019. Pregnant women and their partners were contacted by a researcher for informed consent. Inclusion criteria were pregnant women and men with pregnant partners expecting their first child and living in one of the five city districts of Oslo. The exclusion criteria were multiparous women.

We were not allowed to collect background information on participants who were unwilling to participate. Thus, selection bias is possible if our respondents differ from non-respondents. The number of respondents eligible and willing to participate during the study period determined the sample size.

Self-reported measures were sent to the participants by mail. The consent form and all measures were available in ten languages (Norwegian, English, Arabic, Lithuanian, Pashto, Polish, Somali, Tamil, Turkish, and Urdu). All data were returned by the end of January 2020.

### Measures

#### Demographics

Standard demographic data were measured, including family income, educational level, age, nationality, and marital status. In addition, hours of sleep and previous and present mental illness were measured.

#### Outcome

Quality of life was measured by the World Health Organization Quality of Life Questionnaire Brief version (WHOQOL-BREF) [28]. The instrument contains 26 items, which includes one item from each of the 24 facets of WHOQOL-100 and two single items on overall QoL and general health satisfaction. The single items are examined separately, and the remaining 24 items produce the four dimensions physical health (7 items), psychological (6 items), social relationship (3 items), and environment (8 items). All items are assessed on a 5-point Likert scale (range 1–5), with various scale responses. Higher values indicate higher QoL. The domain scores, ranging from 4–20, were calculated by multiplying the mean score of each domain by four, according to the WHOQOL-BREF scoring manual [28]. WHOQOL-BREF is validated in the general Norwegian population [29, 30]. The instrument’s psychometric properties have been reported for both pregnant women and men with pregnant partners [31]. In our study, Cronbach’s alpha for the WHOQOL-BREF domains physical health, psychological, social relationship, and environment were, respectively, 0.80, 0.81, 0.67, and 0.78 for the pregnant women and 0.74, 0.85, 0.54, and 0.72 for the men with pregnant partners.

#### Selective possible predictive factors

Perception of sleep was measured by a single item: Do you feel that you get enough sleep (Yes/No)? Complications during pregnancy were also measured by a single item: Did you have any complications during your pregnancy (Yes/No)?

The Edinburgh Postnatal Depression Scale (EPDS) [32] was used to assess depressive symptoms. Respondents are asked about symptoms in the past seven days. The EPDS contains ten items scored on a 4-point Likert scale, ranging from 0 to 3. The total score of the scale ranges from 0–30, with higher scores indicating higher levels of depressive symptoms. A cut-off score of 10, where < 10 indicates no depressive symptoms and ≥ 10 indicates depressive symptoms. The EPDS has been validated for postnatal use in women [33] in the Norwegian population. Validation studies have been conducted with pregnant women [34–36] but not men in international studies. In our study, Cronbach’s alpha for the total scale of EPDS was 0.83 for the pregnant women and 0.74 for the men with pregnant partners.

### Statistical analyses

Sample characteristics were described separately for pregnant women and men with pregnant partners using descriptive statistics. Categorical data were presented as counts and percentages, and continuous variables were described as means and standard deviation (SD). Crude comparisons of the pregnant women and men with pregnant partners on background variables were performed using t-test for continuous variables and chi-square for categorical variables. As the level of education and family income were highly correlated, we used the level of education only as a possible predictive factor in all analyses to avoid multicollinearity. The number of missing values for each item is presented.

Descriptive analyses were used to determine the WHOQOL-BREF single items and domain scores in pregnant women and men with pregnant partners, described by mean and SD. Chi-square test was used to determine the association between pregnant women and men with pregnant partners on the two WHOQOL-BREF single items, described by p-value. Independent sample t-test was used to determine the differences in WHOQOL-BREF domain scores between pregnant women and men with pregnant partners, described by mean difference, 95% confidence intervals (95% CI), and p-value.

Moderation analyses were used to explore the relationship between the four dependent variables of WHOQOL-BREF domain scores and the independent variable of perception of sleep and the possible moderating effect of depressive symptoms. Moderation analyses were performed as described by Hayes [37], using the PROCESS macro with model 1. The results are presented with unstandardized coefficients (B) and 95% CI. All the confidence intervals were derived using bootstrapping with 5000 repetitions. Age, pregnancy week, complications during pregnancy, and educational level were included as confounders in the model. Furthermore, multivariate linear regression was used in the sub-analysis of pregnant women to investigate if the selected predictive factors were statistically significantly associated with the WHOQOL-BREF domain scores when stratified by depressive symptoms. Educational level was treated as an ordinal variable with three levels in all regression analyses. All other variables were continuous or dichotomous. Internal consistency reliability was examined by calculating Cronbach’s alpha for all four domain scales of WHOQOL-BREF and the total scale of EPDS.

All statistical analyses were conducted in SPSS, version 28, in the secure platform of Services for Sensitive Data [38]. The level of statistical significance was set to *p* < 0.05 for all analyses, and all point estimates are reported with 95% CI.

## Results

**Fig. 1:**
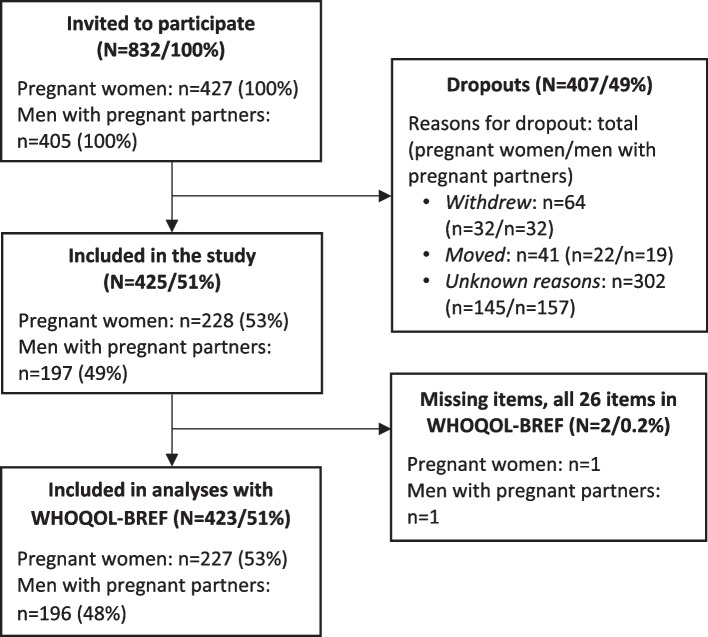
Flow chart on study sample

In this study, 228 pregnant women and 197 men with pregnant partners were included. A flow chart of the study sample is presented in Fig. 1. Two participants did not answer the WHOQOL-BREF and were excluded from the analyses. Participants completed the measures in three languages: Norwegian used by 218 (95.6%) pregnant women and 186 (94.4%) men with pregnant partners, English by eight (3.5%) pregnant women and eight (4.1%) men with pregnant partners, and Arabic by one (0.4%) pregnant woman and two (1.0%) men with pregnant partners.

The majority of the participants were between 29–35 years, partnered or married, achieved a high educational level, and were Norwegians (Table 1). A statistically significantly higher proportion of pregnant women compared to men with pregnant partners reported not getting enough sleep (37.7% and 23.4%) and having depressive symptoms (17.9% and 3.1%). The majority of the sample reported no history of mental illness, but the pregnant women had statistically significantly higher frequency of previous mental illness.

**Table 1:** Sample characteristics of pregnant women and male partners

	**Pregnant women (*N* = 228)**				**Men with pregnant partners (*N* = 197)**				**Comparison**	
	**n (missing)**	**%**	**Mean (min–max)**	**SD**	**n (missing)**	**%**	**Mean (min–max)**	**SD**	***p***	**95% CI**
**Age**	228 (0)	100	31.38 (22–47)	3.98	195 (2)	99.0	33.08 (22–50)	4.83	** < 0.001**	**-2.55 to -0.84**
< 28	46	20.2			30	15.2				
29–35	149	65.4			119	60.4				
36 <	33	14.5			46	23.4				
**Pregnancy week**	222 (6)	97.4	31.73 (26–40)	3.60	193 (4)	98.0	31.79 (26–41)	3.54	0.869	-0.75 to 0.63
Week 26–30	104	45.6			86	43.7				
Week 31–35	74	32.5			73	37.1				
Week 36–41	44	19.3			34	17.3				
**Marital status**	217 (11)	95.2			196 (1)	99.5			NA^a^	
Single	7	3.1			1	0.5				
Partnered	130	57.0			122	61.9				
Married	80	35.1			73	37.1				
**Educational level**	227 (1)	99.6			196 (1)	99.5			**0.025**	
Primary/secondary school	22	9.6			34	17.3				
College/university (< 4 years)	65	28.5			63	32.0				
College/university (≥ 4 years)	140	61.4			99	50.3				
**Family income, before tax (NOK)**	222 (6)	97.4			192 (5)	97.5			NA^a^	
< 750.000	36	15.8			30	15.2				
750.000–1.000.000	67	29.4			53	26.9				
> 1.000.000	119	52.2			109	52.2				
**Nationality**	228	100			196 (1)	99.5			**0.034**	
Norwegian	196	86.0			152	77.2				
Other	32	14.0			44	22.3				
**Complications during pregnancy**	226 (2)	99.1			NA^a^				NA^a^	
Yes	53	23.2								
**Depressive symptoms**^**b**^	224 (4)	98.2			193 (4)	98.0			** < 0.001**	
Yes	40	17.9			6	3.1				
**Mental illness**	227 (1)	99.6			196 (1)	99.5				
Present	9	3.9			6	3.0			0.812	
Previous	30	13.2			10	5.1			**0.007**	
**Sleep, hours**	216 (12)	94.7	7.36 (3–12)	1.32	180 (17)	91.4	6.95 (4–9)	0.85	** < 0.001**	**0.19 to 0.62**
**Sleep**	224 (4)	98.2			193	98.0			**0.002**	
Enough	138	60.5			147	74.6				
Not enough	86	37.7			46	23.4				

^a^*NA* Not Assessed

^b^Measured by *EPDS* cutoff for depressive symptoms ≤ 10

### Quality of life

The overall QoL (WHOQOL-BREF single item on QoL) was rated to be good/very good by 215 of the pregnant women (95.5%) and 188 of the men with pregnant partners (96.5%). One hundred and eighty-nine of the pregnant women (84%) and 168 of the men with pregnant partners (86.1%) reported that they were satisfied/very satisfied with their general health (WHOQOL-BREF single item on health satisfaction). There were no statistically significant differences between the two groups. For the physical health and psychological domains of the WHOQOL-BREF, the pregnant women reported statistically significantly lower scores than the men with pregnant partners (Table 2).

**Table 2:** WHOQOL-BREF domain scores (physical health, psychological, social relationship, environment) in pregnant women and men with pregnant partners

QoL domains	Men with pregnant partners (*n* = 196)		Pregnant women (*n* = 227)		Comparison		
	**Mean**	**SD**	**Mean**	**SD**	**Mean difference**	**95% CI**	***p*-value**
**Physical health**	17.00	1.89	14.86	2.51	**-2.14**	**-2.56 to -1.72**	** < 0.001**
**Psychological**	16.23	2.41	15.68	2.20	**-0.55**	**-0.99 to -0.11**	**0.015**
**Social relationships**	15.70	2.30	15.41	2.54	-0.27	-0.74 to 0.19	0.248
**Environment**	17.12	1.77	16.81	1.94	-0.31	-0.66 to 0.05	0.093

### Quality of life, enough sleep, and depressive symptoms

In total, 213 pregnant women answered all the included items in the moderation model. A relationship between the perception of sleep on the four domains of QoL was not moderated through depressive symptoms (Table 3). Depressive symptoms were a statistically significant predictor of poorer QoL in all four WHOQOL-BREF domains. Our data revealed only an additive effect of both getting enough sleep and depressive symptoms, as the effect of sleep was not moderated by the level of depressive symptoms (all interaction terms sleepXdepressive symptoms had *p* < 0.2). Those who reported getting enough sleep had on average 1.5 higher scores on the physical health domain compared to those who did not get enough sleep (B = 1.49, 95%CI [0.55 to 2.33]). Those who reported having complications during pregnancy had on average 1.2 lower scores on the physical health domain compared to those who did not report complications (B = -1.24, 95%CI [-2.04 to -0.43]). Higher educational level was associated with higher QoL scores in both the physical health (B = 0.65, 95%CI [0.26 to 1.07]) and environmental domain (B = 0.61, 95%CI [0.18 to 1.06]).

**Table 3:** Moderation analysis in pregnant women (*n* = 213), of the relationship between the perception of sleep and WHOQOL-BREF domains and depressive symptom as a moderator

	**Physical health**		**Psychological**		**Social relationships**		**Environment**	
Moderation model	**B**	**95% CI**	**B**	**95% CI**	**B**	**95% CI**	**B**	**95% CI**
Perception of sleep (ref = not having enough sleep)	**1.49**	**0.55 to 2.33**	0.44	-0.32 to 1.20	0.60	-0.34 to 1.49	0.28	-0.46 to 0.86
Depressive symptoms (ref = EPDS < 10)	**-1.94**	**-2.87 to** **-1.12**	**-2.82**	**-3.56 to -2.08**	**-1.81**	**-2.73 to -0.93**	**-1.36**	**-2.03 to -0.71**
Enough sleep x depressive symptoms	0.68	-1.21 to 2.33	0.55	-0.99 to 2.07	1.16	-0.75 to 2.92	-0.11	-1.49 to 1.29
Confounders:								
Age	0.05	-0.03 to 0.12	-0.03	-0.09 to 0.03	-0.07	-0.15 to 0.01	-0.03	-0.10 to 0.04
Pregnancy week	-0.06	-0.14 to 0.02	0.04	-0.02 to 0.11	0.05	-0.04 to 0.14	0.02	-0.05 to 0.09
Complications during pregnancy	**-1.24**	**-2.04 to** **-0.43**	-0.27	-0.84 to 0.32	-0.42	-1.24 to 0.44	-0.26	-0.82 to 0.30
Educational level (three levels)	**0.65**	**0.26 to 1.07**	0.11	-0.26 to 0.48	0.02	-0.52 to 0.49	**0.61**	**0.18 to 1.06**

We did not conduct moderation analyses on men with pregnant partners, as only six men were categorized with depressive symptoms. We analyzed the association between the QoL domains and perception of sleep, controlling for age, the woman’s pregnancy week, and educational level. Getting enough sleep was statistically significantly associated with higher QoL domain scores: physical health (B = 1.98, 95% CI [1.45 to 2.55]), psychological (B = 1.43, 95% CI [0.63 to 2.22]), social relationship (B = 1.08, 95% CI [0.31 to 1.85]), and environment (B = 0.74, 95% CI [0.17 to 1.30]).

### Women with and without depressive symptoms

Analysis of pregnant women stratified by depressive symptoms was conducted to understand more about depressive symptoms as a statistically significant predictor of the WHOQOL-BREF domains revealed in the moderation analysis. The pregnant women without depressive symptoms had statistically significantly higher QoL domain scores than the pregnant women with depressive symptoms (Table 4).

**Table 4:** WHOQOL-BREF domain scores (physical health, psychological, social relationship, environment) in stratified analyses of pregnant women with (*n *= 40) and without (*n* = 178) depressive symptoms

QoL domains	Depressive symptoms (*n* = 40)		Without depressive symptoms (*n* = 184)		Comparison		
	**Mean**	**SD**	**Mean**	**SD**	**Mean difference**	**95% CI**	***p*-value**
**Physical health**	12.83	2.47	15.31	2.32	**2.48**	**1.68 to 3.29**	** < 0.001**
**Psychological**	13.20	2.29	16.22	1.78	**3.02**	**2.24 to 3.79**	** < 0.001**
**Social relationships**	13.70	2.73	15.75	2.34	**2.05**	**1.22 to 2.88**	** < 0.001**
**Environment**	15.59	1.94	17.07	1.85	**1.49**	**0.85 to 2.13**	** < 0.001**

Table 5 shows that in the stratified group of pregnant women without depressive symptoms (*n* = 178), getting enough sleep and educational level were associated with higher QoL scores in the physical health domain (B = 1.23, 95% CI [0.63 to 1.85] and B = 0.82, 95% CI [0.44 to 1.20] respectively) and complications during pregnancy with lower QoL scores on the physical health domain (B = -1.02, 95% CI [-2.27 to -0.34]). Additionally, educational level was associated with higher QoL scores in the environment domain (B = 0.65, 95% CI [0.10 to 1.28]).

**Table 5:** Stratified analyses of WHOQOL-BREF domain scores in pregnant women without (*n* = 178) and with depressive symptoms (*n* = 40), and association with enough sleep, complications during pregnancy and educational level

	**Depressive symptoms (*n* = 40)**			**Without depressive symptoms (*n* = 178)**		
	**B**	**95% CI**	***p*-value**	**B**	**95% CI**	***p*-value**
**Physical health**						
Enough sleep	1.66	-0.36 to 2.86	0.078	**1.23**	**0.63 to 1.85**	** < 0.001**
Complications during pregnancy	-1.24	-2.60 to 0.43	0.176	**-1.02**	**-2.27 to -0.34**	**0.036**
Education level	0.18	-0.75 to 1.04	0.748	**0.82**	**0.44 to 1.20**	** < 0.001**
**Psychological**						
Enough sleep	0.81	-1.15 to 2.63	0.268	0.24	-0.55 to 0.93	0.398
Complications during pregnancy	-0.75	-2.95 to 1.33	0.359	0.01	-0.48 to 0.47	0.976
Education level	-0.47	-1.31 to 0.28	0.340	0.29	0.01 to 0.63	0.146
**Social relationships**						
Enough sleep	1.43	-0.89 to 3.60	0.123	0.17	-0.92 to 1.22	0.657
Complications during pregnancy	-0.99	-2.48 to 0.46	0.321	-0.15	-1.78 to 1.20	0.727
Education level	-0.87	-2.85 to 1.19	0.254	0.10	-0.28 to 0.50	0.687
**Environment**						
Enough sleep	0.27	-0.75 to 1.17	0.672	0.37	-0.50 to 1.38	0.229
Complications during pregnancy	-0.36	-2.31 to 1.93	0.631	-0.08	-0.55 to 0.41	0.822
Education level	0.61	-0.21 to 1.40	0.277	**0.65**	**0.10 to 1.28**	**0.006**

## Discussion

The key findings of our study were that the pregnant women experienced diminished QoL compared to the men with pregnant partners and that the pregnant women with depressive symptoms had statistically significantly lower QoL compared to the pregnant women without depressive symptoms. Finally, the perception of getting enough sleep was statistically significantly associated with better QoL in all domains in the men with pregnant partners and the physical health domain in the pregnant women without depressive symptoms.

The pregnant women in our study showed statistically significantly lower scores in the physical health and psychological domains compared to the men with pregnant partners. For physical domains measured during late pregnancy, similar results have been reported on differences between pregnant women and men with pregnant partners [12, 13]. In the pregnant women, the physical health domain was the most obviously affected, in line with previous findings [8, 9]. In the group of pregnant women without depressive symptoms, physical health was the only domain score that remained low compared to the men with pregnant partners. For psychological domains, QoL scores in pregnant women have been shown to be both lower [13] and higher [12] compared to men with pregnant partners. The pregnant women without depressive symptoms in our study demonstrated nearly similar scores as the men with pregnant partners in the psychological domain. This can be seen in relation to previous results, showing that psychological domains remain stable and even increases in women throughout pregnancy [8]. Our result confirms that the QoL on the psychological domain is high in mentally healthy pregnant women and men with pregnant partners during the third trimester of pregnancy. The men with pregnant partners reported good QoL on all domains compared to the pregnant women in our study.

The ratings on the two single items general health and overall QoL were similarly high in the pregnant women and the men with pregnant partners in our study. Despite lower domain scores, the presence of depressive symptoms, pregnancy complications, and inadequate sleep in our sample of pregnant women, the majority reported satisfaction with their general health and overall QoL. Similar results regarding high perceived general health and lower domain scores are previously reported in pregnant women [10]. Our results could be seen in relation to WHOs description of the state of health as “not merely the absence of disease or infirmity” [7], but also to its assertion that individuals’ perception of their QoL develops in relation to their goals and expectations [5]. One explanation for the results might be the multidimensional construct of QoL [4] and the theory of response shift [39]. An individual’s self-evaluation of the importance of a component in the target construct may change between the domains and the single items [39]. In a salutogenic perspective, the focus is on the origin of health and on enhancing individuals well-being by utilizing internal and external protective factors [40]. From this perspective, our results could imply that the general health and overall QoL of the pregnant women is enhanced by their ability to utilize the resources available and thereby their ability to experience their life situation as comprehensive and meaningful [41]. A strong capacity to use available resources has been identified as a predictor of well-being in pregnant women [42]. Midwives and PHNs in prenatal care may support pregnant women during pregnancy, especially by identifying and acknowledging pregnancy-related challenges, providing resources, or enabling them to utilize their assets.

The results of our study showed that the physical health domain was statistically significantly associated with the perception of getting enough sleep, complications during pregnancy and educational level. These factors remained statistically significantly associated in the stratified group of pregnant women without depressive symptoms. Higher QoL on the physical health domain was statistically significantly associated with getting enough sleep, in line with previous findings [10, 16]. The perception of sleep was not associated with the other QoL domains. This may imply that the physical changes and challenges during late pregnancy in our sample of pregnant women affected their sleep. This interpretation is strengthened by our finding of a statistically significantly association between low QoL on the physical domain and complications during pregnancy, in line with previous studies [8, 9]. These results imply that the prenatal care may address and evaluate challenges of sleep and complications during pregnancy and provide professional support to improve the physical health QoL outcomes of pregnant women. The finding of a statistically significantly association between higher educational level and higher QoL scores on the physical health domain, as reported in previous studies [10, 16], is a demographic factor clinicians in prenatal care need to be aware of in individuals.

In the men with pregnant partners, the perception of getting enough sleep during pregnancy was found to be a statistically significant predictor of higher scores on all QoL domains. Contrarily, in the pregnant women, this association was only found statistically significant on the physical health domain. These results may imply that the perception of enough sleep is more important for the QoL in men with pregnant partners during the period of pregnancy compared to pregnant women, or that it is more important for the QoL of men in general. Poor sleep quality as a predictor of reduced QoL in the general population of men has previously been documented [43]. Further research is needed to understand more about the importance of sleep for men with pregnant partners, preferably in association with sleep in pregnant women.

Depressive symptoms were a statistically significant predictor of lower scores on all QoL domains in the pregnant women in our study, with the group of pregnant women with depressive symptoms showing diminished QoL domain scores, in line with previous findings [10, 16, 21, 22]. In health care, individuals QoL outcomes are most commonly measured through physical, psychological and social dimensions [3, 4]. Thus, the pregnant women with depressive symptoms in our study are affected on all crucial aspects of QoL related to health. This supports the need for clinicians in the prenatal care to identify these women and contribute to provide them appropriate treatment.

In the group of pregnant women with depressive symptoms, we did not find any statistically significant association between the QoL domain scores and the selected possible predictive factors. However, we see similarities when comparing the regression coefficients (B) of factors in the groups of pregnant women with and without depressive symptoms. This implies that perceptions of getting enough sleep and complications during pregnancy are factors influencing the physical health QoL of pregnant women with depressive symptoms. The lack of statistical significance in this group may be due to lack of statistical power, as the group were represented by 40 individuals. Previously, low QoL attributed to physical domains in pregnant women with depressive symptoms have been found associated with both sleep problems [10, 16] and complications during pregnancy [10]. Depressive symptoms in pregnancy are likely to continue into the postpartum period [19] and maternal depression is found to be a moderator of paternal depression [20]. Hence, this supports the importance of identifying and addressing depressive symptoms in the prenatal care.

### Strengths and limitations

A strength of our study was the examination of QoL for both pregnant Norwegian women and men with pregnant partners. Our data did not reveal any moderating effect of depressive symptoms on the association between getting enough sleep and QoL. However, the lack of statistically significant interaction might be due to a limited statistical power as there were only 40 women who reported having depressive symptoms. Thus, we cannot rule out that not getting enough sleep might have an additional negative effect on QoL for those with depressive symptoms and further larger studies are warranted.

The study had some limitations, including homogeneity (well-educated and high-income), which may limit generalizability. The WHOQOL-BREF is a generic QoL instrument that does not measure pregnancy-related factors. The study was cross-sectional and did not address changes in QoL or the cause of changes in QoL. Thus, our data cannot determine the effect of pregnancy on the QoL or other factors in our sample, as we do not have pre-pregnancy data. Several factors affect an individual’s sleep or the chance of developing depressive symptoms.

## Conclusions

Women in the final trimester of pregnancy experience poor QoL compared to men with pregnant partners. Our data did not support that depressive symptoms moderated the relationship between the perception of sleep and QoL. It must be explored in a larger sample of pregnant women and men with pregnant partners for further testing. Pregnant women with depressive symptoms have lower QoL compared to those without depressive symptoms. The perception of getting enough sleep was statistically significantly associated with better QoL in all domains in the men with pregnant partners and in the physical health domain in the pregnant women.

## Data Availability

The data used and analyzed in this study are available from the corresponding author upon reasonable request.

## Abbreviations

QoL: Quality of Life
WHO: World Health Organization
NF: New Families (name of home visiting program/research project)
PHN: Public Health Nurse
WHOQOL-BREF: World Health Organization Quality of life Questionnaire Brief version
EPDS: Edinburgh Postnatal depression Scale
SD: Standard Deviation
CI: Confidence Interval
